# How to Implement Pressurized Intraperitoneal Aerosol Chemotherapy into a National Health System Scenario: A Single-Center Retrospective Analysis of Costs and Economic Sustainability at a High-Volume Italian Hospital

**DOI:** 10.3390/cancers16152637

**Published:** 2024-07-24

**Authors:** Matteo Aulicino, Cecilia Orsini, Giorgio D’Annibale, Lorenzo Barberis, Paolo Catania, Carlo Abatini, Miriam Attalla El Halabieh, Federica Ferracci, Claudio Lodoli, Francesco Santullo, Fabio Pacelli, Andrea Di Giorgio

**Affiliations:** 1 General Surgery Department, Università Cattolica del Sacro Cuore, 00168 Rome, Italy; 2 Surgical Unit of Peritoneum and Retroperitoneum Surgery, Fondazione Policlinico Universitario Agostino Gemelli IRCCS, 00168 Rome, Italy

**Keywords:** surgical oncology, PIPAC, peritoneal carcinomatosis, operating profit, National Health System reimbursement

## Abstract

**Simple Summary:**

Pressurized intraperitoneal aerosol chemotherapy (PIPAC) is a new surgical procedure used in the treatment of peritoneal surface malignancies (PSMs). Despite encouraging clinical results, the high costs of PIPAC and the need to repeat the treatment multiple times make the procedure difficult to sustain economically. A retrospective study was conducted in order to evaluate the cost of hospitalization for patients undergoing PIPAC treatment at a high-volume center in Italy. Currently, the Italian National Health System only partially covers the hospitals’ costs. Therefore, there is an urgent need for an economical redefinition of this procedure, by creating dedicated reimbursement codes.

**Abstract:**

PIPAC is a new surgical procedure and a viable treatment option for PSM patients, due to promising therapeutic outcomes, minimal invasiveness, limited surgical morbidity, and systemic toxicity side effects. However, its implementation throughout hospitals is hard to obtain due to its fragile economical sustainability. A retrospective health economic analysis was conducted in order to evaluate the cost of hospitalization for patients undergoing PIPAC treatment at Fondazione Policlinico Universitario Agostino Gemelli, IRCCS, in Rome. The average cost of a PIPAC procedure was defined based on the cost of surgery (cost of surgical material, operating room, intraperitoneal chemotherapy), hospital stay, diagnostic examinations, and drugs used during the stay. A total of 493 PIPAC procedures were performed on 222 patients with peritoneal metastases or primary peritoneal cancer from 2017 to 2023. Since the mean remuneration for each PIPAC hospitalization is €5916 and the mean expenditure per hospitalization is €6538, this results in an operating profit per PIPAC hospitalization of −€622. The reimbursement of PIPAC treatment by the Italian National Health System currently only partially covers the hospital’s costs. Development of specific codes and adequate reimbursement for PIPAC by recognizing this procedure as a proper treatment for peritoneal carcinomatosis is essential.

## 1. Introduction

Pressurized intraperitoneal aerosol chemotherapy (PIPAC) is a new surgical procedure for the treatment of peritoneal surface malignancies (PSMs), first described in 2011. Most patients have low life expectancy and poor quality of life, characterized by abdominal pain, bowel obstruction, and ascites [1]. Until recent times, the only possible treatment for subjects who were not eligible for cytoreductive surgery has been systemic chemotherapy [2,3,4,5]. Compared to other metastatic sites, systemic chemotherapy is less effective for peritoneal metastases because the reduced blood supply and high interstitial pressure prevent systemic treatments from reaching and targeting tumor cells. PIPAC increases drug concentrations in targeted peritoneal tissues while maintaining low plasma concentrations, thus reducing the risk of systemic toxicity [6,7,8]. The combination of PIPAC and systemic chemotherapy has been shown to provide better survival, preserve and, in some cases, to improve the quality of life (QOL) [9,10,11].

Despite the encouraging preliminary clinical results [12,13,14,15], the high costs of PIPAC and the need to repeat treatment multiple times make it a procedure difficult to sustain economically. Moreover, since PIPAC is a relatively recent treatment and performed in a limited number of referral centers, ICD (International Classification of Diseases) and DRGs (Diagnosis-Related Groups) codes are not available in Italy for correct coding of and reimbursement for the procedure by the Italian National Health System (NHS). To our knowledge, no study has been conducted to evaluate the costs of this treatment in Italian hospitals. The aim of this work is to evaluate the average cost of hospitalization and the average hospital reimbursement, and finally to define the economical sustainability of the procedure.

## 2. Materials and Methods

### 2.1. Economical Analysis

A health economic analysis was carried out in order to evaluate the cost of hospitalization for patients undergoing PIPAC treatment at a high-volume center. Patients with peritoneal carcinomatosis from different primary tumors undergoing PIPAC at Gemelli Hospital between January 2017 and August 2023 were selected. Since a specific procedure code does not exist yet, the collection of data was performed by a combined search of existing intervention codes, derived from the International Classification of Diseases (i.e., ICD) coding system, used in PIPAC surgical reports (laparoscopy: ICD9-CM code 54.21; injection or infusion of chemotherapy substances for cancer: ICD9-CM code 99.25; injection of therapeutic substances with local action into the peritoneal cavity: ICD9-CM code 54.97). Although newer versions are available, the ICD9-CM coding system was chosen because it is currently in use in Italy.

The average cost of a PIPAC procedure was calculated as the average of money billed by the hospital at each hospitalization to cover the cost of surgery (cost of surgical material, operating room, intraperitoneal chemotherapy), hospital stay (hospitalization, pre- and post-operative management expenses, hourly costs of medical personnel), diagnostic examinations, and drugs used during the stay. Routine diagnostic examinations include a pathology workup of peritoneal biopsies and cytology tests performed on peritoneal washings. In case of PIPAC associated with complications, when performed, imaging studies, microbiological investigations, or interventional acts were added up to the final amount. The medical staff cost (2 surgeons, 1 anesthesiologist, and 2 nurses) is included in the total cost of the operating room. Cost details for each PIPAC procedure were provided by the Accounting Office of Fondazione Policlinico Universitario Agostino Gemelli.

Average length of stay (calculated from the day of admission to the day of discharge), mean total operating room occupancy time, and type of chemotherapy performed during PIPAC also had to be accounted for.

The injection system used for PIPAC procedures is an Angio Injector that was already available at Gemelli Hospital for vascular procedures. Therefore, there was no need to purchase a separate injection system for PIPAC procedures, and it did not result in additional costs.

It was not possible to estimate pharmacy charges due to a complex billing system that does not allow access to individual costs per procedure.

The hospital remuneration for each PIPAC depends on how the hospitalization is classified according to the Diagnosis-Related Groups (DRGs) classification. Each DRG can be modified by the presence of complications, additional procedures, or concomitant diagnoses.

The hospital operating profit was then calculated as the difference between the average reimbursement and the average cost for performing each PIPAC procedure from January 2017 to November 2023.

### 2.2. Surgical Procedure

The procedure adhered to current safety recommendations and protocols [16,17,18,19]. Under general anesthesia, a 10–12 mm laparoscopic balloon trocar is placed through the open laparoscopic access technique, and a 12 mmHg capnoperitoneum at 37 °C is created. Then, one or two 5 mm laparoscopic balloon trocars are placed. During the procedure, the presence of ascites is documented, paracentesis is performed, and a sample is sent for cytology; a thorough laparoscopic exploration is performed in order to calculate PCI and assess for involvement of other organs. Finally, multiple biopsies are performed in the different abdominal quadrants. PIPAC treatment involves the delivery of a pressurized aerosol through the use of a CAPNOPEN^©^ nebulizer (Capnopharm GmbH, Tübingen, Germany) connected to a high-pressure injector that is inserted inside the 10–12 mm trocar at a 45° angle, in order to promote better diffusion of the chemotherapy within the abdominal cavity.

The system is then kept in steady state for 30 min (application time) and the residual toxic aerosol is then expelled via a closed surgical smoke evacuation system, thus avoiding its dispersal in the external environment. Finally, trocars are extracted and laparoscopy is terminated without the need for placement of abdominal drains.

Chemotherapy combinations used in our study are consistent with those reported in the literature: oxaliplatin 92 mg/m^2^ was used for peritoneal metastases from colorectal and appendiceal carcinoma, and for pseudomyxoma peritonei. The combination of cisplatin 7.5 mg/m^2^ + doxorubicin 1.5 mg/m^2^ was used for peritoneal gastric, ovarian, pancreatic, and biliary tract metastases, and for mesothelioma. Since 2018, a higher dose of cisplatin and doxorubicin (10.5 and 2.1 mg/m^2^, respectively) has been adopted after the Tempfer et al. phase I study. In addition, the use of nab-paclitaxel 112.5 mg/m^2^ has been introduced since March 2023 for patients with peritoneal carcinomatosis from pancreatic cancer, in the context of the Nab-PIPAC phase II controlled trial to evaluate the efficacy of this drug [11,12,13,14,15,16,17,18,19,20].

### 2.3. Statistical Analysis

Continuous variables were reported as mean ± standard deviation (SD). Categorical variables were reported as absolute and relative frequencies (%). Statistical analysis and graph processing were performed using GraphPad Prism 9, the IBM SPSS software v29.0.1.0 platform, and Excel v16.54.

## 3. Results

A total of 493 PIPAC procedures were performed on 222 patients with peritoneal surface malignancies (PSMs). The specific distribution of PIPAC procedures according to primitive tumor location are summarized in Table 1.

Chemotherapy regimens were distributed as follows: cisplatin and doxorubicin in 366 patients (74%), oxaliplatin in 67 patients (14%), and nab-paclitaxel in 60 patients (12%). Mean drug cost was 28 ± 28,389 € per procedure.

Mean length of stay was 2.3 ± 1.23 days with a mean expense of 1380 ± 271 € per patient.

Mean operating room occupation time was 123.26 ± 39.14 min, with an average cost of 1156 ± 286 € per procedure.

Mean in-hospital diagnostic and therapeutic procedures expenses were 440 ± 190 €.

Average device and surgical tools cost was 3534 €, with the PIPAC kit accounting for the largest sum of it (€3330).

Total mean hospitalization expense per PIPAC was estimated to be 6538 ± 663 €.

PIPAC procedures were mainly classified with two DRG codes: DRG 171 (“Other digestive tract procedures without complications”) and DRG 170 (“Other digestive tract procedures with complications”). DRG code number 171 was assigned to 315 (64%) patients, while DRG 170 was assigned to 148 (30%) patients. Hospital-fixed-reimbursement related to DRG 171 and DRG 170 was €4498 and €8810, respectively, for each PIPAC. The hospital operating profit for each PIPAC was −€2040 for DRG 171 and 2272 € for DRG 170. Thirty patients (6%) were given a DRG code different than the abovementioned due to an associated procedure (DRG 357: “Operations on the uterus and adnexa for malignant neoplasms of the ovary or appendages”; DRG 355: “Operations on the uterus and adnexa for malignant neoplasms not of the ovary or appendages”).

Concerning the total PIPAC procedures performed, the mean hospital reimbursement was 5916 ± 1422 € and the mean operating profit for each PIPAC was –€622. Total Gemelli-hospital PIPAC costs not covered by the Italian National Health System from January 2017 to November 2023 were 306,646 €. The summary of expenses is shown in Table 2.

## 4. Discussion

In this study, we conducted a medical economic analysis in order to assess the financial sustainability of the PIPAC procedure in a high-volume center such as Fondazione Policlinico Universitario Agostino Gemelli, IRCCS. Since there is not a specific codification system of PIPAC according to the current ICD, PIPAC procedures are identifiable by a combination of different existing intervention codes. This makes PIPAC coding an operator-dependent procedure, resulting in high discrepancies in medical record codification in the same center and among different institutions, with implications on the reimbursement by the National Health System. In a study conducted by Fatah Tidadini et al. [21] comparing the reimbursement of two French hospitals, a median difference of 853 € (€4476 vs. €3623) per hospitalization was documented. Although PSMs are recognized as a specific nosological entity, the ICD9-CM does not include surgical procedures to treat this condition. Consequently, the intraperitoneal surgical delivery of chemotherapy trough hyperthermic intraperitoneal chemotherapy (HIPEC) and PIPAC procedures are both actually codified with an aspecific code (ICD9-CM 99.25), like the one used for systemic chemotherapy. Therefore, a specific procedural code for PIPAC is mandatory.

For the same reason, the absence of a specific DRG code for PIPAC is very limiting. In our experience, only 6% of admissions were classified with specific DRG codes for gynecologic or gastro-intestinal surgeries, due to the concomitant performance of PIPAC with such procedures. On the other hand, 94% of admissions were archived with a DRG related to non-specific procedures performed on the bowel. The hospitalization was then considered without complications (DRG 171) or with complications (DRG 170), depending on the procedures performed during the hospitalization. For example, placing an abdominal tube to drain ascites or radiological demonstration of the primary tumor changes the DRG from uncomplicate to complicated, making the procedure cost-effective. In fact, the difference for the two DRG codes is €4312 (€4498 vs. €8810) and the hospital operating profit is €2272 or −€2040 with DRG 170 or 171, respectively. In our experience, we have found a DRG 170 (complicated) rate of 30%, despite a complication rate of 4.2% according to the CTCAE classification. This discrepancy further supports the need for improvement in the proper coding of admissions for PIPAC.

The absence of an adequate reimbursement by the NHS is associated with an average cost for each PIPAC hospitalization of €6538. About 54% (€3534) of this expenditure is related to surgical equipment, especially the PIPAC kit, which is currently the most impacting factor on the total cost of hospitalization. Actually, there is only one company that sells the PIPAC kit in Italy, resulting in a higher cost of the devices than in other European countries. For example, in Tidadini et al.’s study [21] the cost is €2485.64. Therefore, the procedure remains a prerogative of high-volume centers specialized in the treatment of peritoneal disease. However, production of the PIPAC kit by an increasing number of companies could reduce prices in the near future, thus making the procedure more economically sustainable. In this regard, comparative studies would be useful to evaluate possible differences in terms of the nebulization capacity and homogeneous distribution of chemotherapy of the different devices [22,23,24].

Operating room occupancy time also has a major impact on the cost of PIPAC hospitalization. Although the application time of nebulized chemotherapy is thirty minutes, the average duration of the procedure in our study is about two hours, in line with the literature [15,25]. Both hospital organization and patients’ characteristics do influence the operating room occupancy time. Indeed, patients often have a history of previous surgeries or have undergone multiple PIPAC treatments with a foreseeable difficulty in accessing the abdominal cavity and the need to perform adequate adhesiolysis.

Therefore, although it may be challenging to minimize the costs related to the duration of the surgical procedure, it could be possible to optimize the length of in-hospital stay. Since the beginning of our experience, hospitalization has been performed on the same day of the surgery, after performing a clinical and laboratory checkup about one week before the scheduled day of the PIPAC treatment. Furthermore, discharge was usually performed the day after the procedure, unless there were alterations in the expected postoperative course. This strategy not only reduced the cost of hospitalization but also reduced the impact of multiple hospitalizations on patients’ quality of life.

On the other hand, the average cost related to intraperitoneal chemotherapy and diagnostic and therapeutic procedures performed during hospitalization is minor. In this regard, it is important to point out that the expense related to the hospital stay may increase in case of clinical complications. However, as PIPAC is a procedure associated with a low rate of complications, if performed in referral centers, their economic impact appears to be rather limited.

Moreover, it should be considered that the cost of the chemotherapy preparation staff and the initial investment for purchase of the injection system are not included in our analysis. Specifically, this cost was not accounted for because the Angio Injector was already available at our institution for vascular procedures. In a study of Javanbakht et al. [26] the infusion pump cost per procedure was calculated to be €173 with an initial investment of €35,000. Therefore, in a hospital planning to start PIPAC treatment, this initial expense has to be considered.

Since the mean remuneration for PIPAC hospitalization is €5916 and the mean expenditure per hospitalization is €6538, the mean operating profit per each PIPAC is −€622. This is associated with a considerable economic loss in the long run, with an investment amounting to €306,646 since 2017 to implement PIPAC treatment at Policlinico Agostino Gemelli.

Anyway, the high costs related to the procedure should not be an obstacle to ensuring that patients with advanced cancer receive the best treatment currently available, especially in centers specialized in the treatment of PSM. PIPAC is an effective treatment by increasing survival, reducing PSM-related symptoms and, in selected cases, inducing a conversion to cytoreductive surgery [13,27,28]. These therapeutic outcomes are related to minimally invasive procedures, low mortality and morbidity, and short hospital stay, which therefore do not compromise the quality of life of patients [13,29,30].

The economic sustainability of PIPAC by the National Health System was also assessed by other studies. In the study of Tidadini et al. [21] published in 2021, it was shown that patients with gastric adenocarcinoma treated with a combination of chemotherapy and PIPAC had a shorter total length of hospitalization than patients treated with chemotherapy alone. A cost-effectiveness analysis study comparing PIPAC treatment and chemotherapy for patients with peritoneal carcinomatosis from gastric cancer was also conducted by Javanbakht et al. [26]. In the second-line palliative setting, PIPAC treatment appears to be dominant and associated with lower costs (£15,082 vs. £36,556) and higher efficacy (mean overall survival 7.44 vs. 4.3; total quality-adjusted life-years per patient 0.45 vs. 0.25), compared with chemotherapy alone. In the first-line palliative setting, an incremental cost-effectiveness ratio (ICER) of £31,868 per quality-adjusted life-year (QALY) gain was estimated, with an 83.6% probability of being cost-effective at £50,000, which is currently the National Institute for Health and Care Excellence accepted cost-effectiveness threshold for end-of-life care. Therefore, despite the high costs, PIPAC seems to be an acceptable cost-effective procedure, reducing the long-term health care expenditures for the treatment of patients with PSMs.

This study is the third one in the literature analyzing the costs and economic sustainability of PIPAC and the only publication on this topic in the Italian experience. Data from the Fondazione Policlinico Universitario Agostino Gemelli, one of the highest-volume centers for PIPAC, quite accurately depicts the economic difficulties to implement these procedures in Italian hospitals.

The main limitation of this study concerns the difficulty of data collection, due to lack of a clear coding system for PIPAC. Moreover, since definitive results regarding survival and quality of life are not currently available, a more accurate cost-effectiveness assessment of PIPAC compared with standard chemotherapy treatment will have to await further confirmation.

## 5. Conclusions

This is a preliminary study that aims to analyze the average costs of performing PIPAC treatments in a single Italian center. PIPAC is a safe and effective treatment that has been used for more than a decade in the treatment of patients with PSMs. It is therefore necessary to recognize specific ICD and DRG codes for an economic redefinition of this procedure by the National Health System. It is an ethical duty to guarantee to every patient all the therapeutic options currently available for the treatment of peritoneal carcinomatosis. Further research may be necessary to enhance the economic sustainability of PIPAC, in order to expand its application to other centers.

## Figures and Tables

**Table 1:** Distribution of PIPAC procedures according to primitive tumor location.

Primary Tumor	N° (%)
Gastric cancer	148 (30%)
Ovarian cancer	139 (28%)
Pancreatic cancer	97 (20%)
Colorectal cancer	59 (12%)
Hepatobiliary cancer	30 (6%)
Other primary tumor	20 (4%)

**Table 2:** Summary of PIPAC costs.

Surgical Material		Cost	Total Cost
PIPAC kit		3330 €	3534 €
Other devices		194 €	
Drugs	Numerosity	Cost per drug	Mean cost
Doxorubicin + cisplatin	366	7 €–13 €	28 ± 28,389 €
Oxaliplatin	67	6 €	
Nab-paclitaxel	60	103.9 €	
Hospitalization	Mean length of stay (day)	Cost per day	Mean cost
	2.3 ± 1.23	600 €	1380 ± 271 €
Operating room	Mean occupancy (min)	Cost per hour	Mean cost
	123.26 ± 39.14	564 €	1156 ± 286 €
Diagnostic test/treatment			Mean cost
			440 ± 190 €
Total mean cost of PIPAC hospitalization			6538 ± 663 €
Mean reimbursement for PIPAC hospitalization			5916 ± 1422 €
Fixed reimbursement according to DRGs 170/171			8810 €/4498 €
Hospital operating profit according to DRGs 170/171			2272 €/−2040 €
Mean hospital operating profit for each PIPAC			−622 €
Hospital PIPAC cost not covered by NHS from 2017 from 2023			−306,646 €

## Data Availability

The raw data supporting the conclusions of this article will be made available by the authors on request.
